# Chylous Ascites: Evaluation and Management

**DOI:** 10.1155/2014/240473

**Published:** 2014-02-03

**Authors:** Said A. Al-Busafi, Peter Ghali, Marc Deschênes, Philip Wong

**Affiliations:** ^1^Hepatology Unit, Department of Gastroenterology, Royal Victoria Hospital, McGill University Health Center, Montreal, QC, Canada; ^2^Department of Medicine, College of Medicine and Health Science, Sultan Qaboos University, P.O. Box 35, 123 Muscat, Oman

## Abstract

Chylous ascites refers to the accumulation of lipid-rich lymph in the peritoneal cavity due to disruption of the lymphatic system secondary to traumatic injury or obstruction. Worldwide, abdominal malignancy, cirrhosis, and tuberculosis are the commonest causes of CA in adults, the latter being most prevalent in developing countries, whereas congenital abnormalities of the lymphatic system and trauma are commonest in children. The presence of a milky, creamy appearing ascitic fluid with triglyceride content above 200 mg/dL is diagnostic, and, in the majority of cases, unless there is a strong suspicion of malignancy, further investigations are not required in patients with cirrhosis. If an underlying cause is identified, targeted therapy is possible, but most cases will be treated conservatively, with dietary support including high-protein and low-fat diets supplemented with medium-chain triglycerides, therapeutic paracentesis, total parenteral nutrition, and somatostatins. Rarely, resistant cases have been treated by transjugular intrahepatic portosystemic shunt, surgical exploration, or peritoneovenous shunt.

## 1. Introduction

Chylous ascites (CA) is an uncommon form of ascites, defined as the leakage of the lipid-rich lymph into the peritoneal cavity [1]. Damage or obstruction to the lymphatic system or one of its tributaries produces ascites with a turbid or milky appearance from the high triglyceride content [1]. Asellius, in 1622, first described the lymphatic system in a dog after observing vessels in the mesentery containing a white milky fluid [2] and, in 1694, Morton reported the first case of CA in a 2-year-old boy who died with tuberculosis [2].

The reported incidence of CA is approximately 1 in 20,000 admissions at a large university-based hospital over 20-year period [3]. However, it is believed that the incidence has increased, probably because of prolonged survival of patients with cancer and more aggressive cardiothoracic and abdominal interventions as well as laparoscopic surgery and transplantation [2]. This trend is supported by the finding of a 1 per 11,589 incidence in the last years of the study [3]. The reported incidence would also probably greatly increase if paracentesis and an appropriate analysis of the ascitic fluid were performed with all patients with ascites [2]. The prognosis basically varies based on the underlying cause. In the same study, the 1-year mortality rate was 71%, which increased to 90% when a malignancy was the underlying cause. Other study that included a greater proportion of congenital or traumatic cases has reported a lower mortality rate (43% in adults and 24% in children) [4]. The mortality becomes even lower in selected groups, such as those with postoperative CA [2].

The aim of this review is to outline the causes of chylous ascites, present a paradigm for investigations, and describe the various management options.

## 2. Anatomy of the Lymphatic System

**Figure 1:**
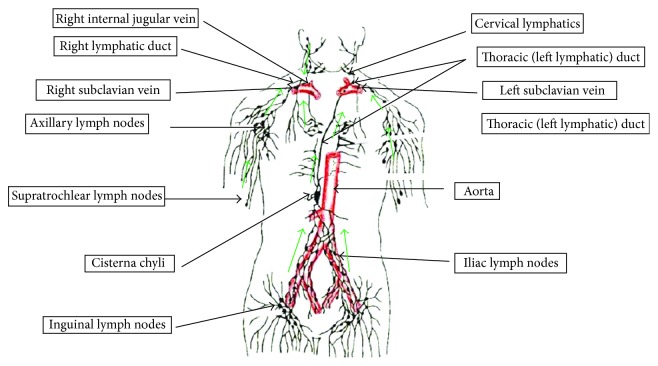
Routes for drainage of lymph from lymph trunks into the thoracic and right lymphatic ducts. The green arrows indicate the direction of lymph flow.

The lymphatic system includes lymph, lymphatic vessels, lymphatic tissues, and red bone marrow (Figure 1) [5]. It is a one-way drainage system which allows the return of excess interstitial fluids and proteins to the vascular system [2]. Lymph passes from lymphatic capillaries into lymphatic vessels and then through lymph nodes into lymph trunks (Figure 1). The thoracic duct, the main duct for the return of lymph to blood, is about 38–45 cm long and begins as dilation called the cisterna chyli anterior to the second lumbar vertebra. The cisterna chyli receives lymph from the right and left lumbar trunks and from the intestinal trunk. Chylous effusions develop when these are injured or obstructed [6].

## 3. Constituents of Chyle 

One of the major functions of the gut lymphatics is the maintenance of the interstitial fluid volume and composition and the transport of lipids. Lymph is composed of protein, lymphocytes, immunoglobulins, and products of digestion including lipids in the form of chylomicrons [7]. More than 50% of the total body lymph originates in the gut and liver [8]. In the gut, long-chain triglycerides (LCT) are converted into monoglycerides and free fatty acids (FFA) and absorbed as chylomicrons. This explains the high content of triglycerides and the milky and cloudy appearance of lymph [9]. Short-and-medium chain triglycerides (MCT), which make up approximately one-third of dietary fat, are absorbed directly by the portal venous system. This particular fact forms the basis for the use of MCT as an oral diet in the conservative management of CA.

Based on animal experiments, Blalock et al. concluded that obstruction of the thoracic duct alone is not sufficient to cause CA [10]. Patients with a limited reserve of anastomotic channels are at greater risk of developing persistent ascites when obstruction or injury of the lymphatic channels occurs.

## 4. Pathophysiology

The principal mechanisms for CA formation are related to disruption of the lymphatic system, from any cause. Three basic mechanisms have been proposed using lymphangiography and inspection at laparotomy [11]:

1. exudation of lymph through the walls of retroperitoneal megalymphatics into the peritoneal cavity, which occurs with or without a visible fistula (i.e., congenital lymphangiectasia),
2. leakage of lymph from the dilated subserosal lymphatics on the bowel wall into the peritoneal cavity which is due to malignant infiltration of the lymph nodes obstructing the flow of lymph from the gut to the cisterna chili,
3. direct leakage of lymph through a lymphoperitoneal fistula associated with retroperitoneal megalymphatics due to acquired lymphatic disruption as a result of trauma or surgery.

In addition, the increased caval and hepatic venous pressures caused by constrictive pericarditis, right-sided heart failure, and dilated cardiomyopathy may precipitate CA through large increase in production of hepatic lymph [12, 13]. Finally, cirrhosis also causes an increased formation of hepatic lymph [14]. In fact, decompression of the portal vein in patients with portal hypertension has been shown to relieve lymphatic hypertension [14–16].

## 5. Etiology

CA may be divided into traumatic and atraumatic causes (Table 1), in which the underlying etiology determines the ongoing evaluation and long-term management. Abdominal malignancy and cirrhosis are the commonest causes in developed countries and account for over two-thirds of all cases, whereas chronic infections like tuberculosis and filariasis account for the majority of the cases in developing countries [1].

### 5.1. Atraumatic CA (Table 1)

In a recent systematic review including 131 studies from developing and developed countries (with a total of 190 patients) who had atraumatic chylous ascites, the most common causes in adults were malignancy (25%), cirrhosis (16%), mycobacterium infection (15%), and a variety of uncommon causes (23%) [17]. In children, the most common causes were lymphatic anomalies (84%) followed by a variety of uncommon causes (15%). Other causes of CA include trauma, including surgical and radiotherapy, and other atraumatic, including congenital, inflammatory, and systemic disorders.

#### 5.1.1. Neoplastic Causes

The most common cause of CA in adults is malignancy. Among the group of malignancies, lymphoma accounts for at least one-third of the cases [17]. Tumors through direct invasion or extrinsic compression lead to disruption of normal lymphatic flow [18]. In addition to lymphomas, other tumors that cause CA may arise from the intra-abdominal solid organ malignancies such as stomach, esophagus, pancreas, endometrial, and prostate, which account for 40% of all malignant causes [17]. Carcinoid tumours and Kaposi sarcoma account for 15% and 9%, respectively, of the malignancy-related cases. Lymphangioleiomyomatosis is a rare benign tumor of lymphatic channels and lymph nodes, clinically manifested by chylous effusions including CA [19].

#### 5.1.2. Congenital

Congenital lymphatic anormalities are predominant cause in the pediatric population, wich accounts for 84% of all causes. In contrast, lymphatic anomalies account only for 9% of atraumatic CA in adults [17]. Primary lymphatic hyperplasia has been recognized as cause of chylous ascites [20]. It consists of two principal patterns: “bilateral hyperplasia” in which the lymphatics are not grossly dilated and contain valves and lymphangiectasia in which lymphatics are grossly dilated in the wall of small bowel and have no valves. The primary intestinal lymphangiectasia is responsible for the majority of the cases in children [17].

Primary lymphatic hypoplasia is another condition seen most commonly in children and presents with lymphedema, chylothorax, CA, or combination [21]. The Klippel-Trénaunay syndrome is an autosomal dominant inherited disorder characterized by venous and lymphatic hypoplastic malformations which can manifest as lower limb lymphedema and it is often associated with chylous ascites [22]. The yellow-nail syndrome is a childhood disorder of unknown etiology. The patients have hypoplastic or aplastic lymphatics leading to the characteristic features of lower limb lymphedema, pleural effusion, and/or CA and a yellow discoloration with dystrophy of the nails [23].

#### 5.1.3. Cirrhosis

Although ascites is a common manifestation of hepatic cirrhosis, CA presents in 0.5–1% of patients with cirrhosis [15, 16, 24]. Recently, a systematic review showed that cirrhosis was responsible for 11% of atraumatic chylous ascites [17]. This discrepancy was explained by the authors to be related to the under diagnosis.

Cirrhotic patients may present with CA as an initial presentation or might present at a later stage of the disease due to complications such as shunt surgery, sclerotherapy-related thoracic duct injury, or hepatocellular carcinoma [24–26]. However, unless clinically indicated, an aggressive approach to exclude malignancy is not warranted. Other cause of CA in cirrhotic patients that should be considered is portal vein thrombosis [27].

#### 5.1.4. Infectious

Lymphatic filariasis and peritoneal tuberculosis are the most common infectious causes of CA and account for the majority of the cases in the developing countries [1]. Low socioeconomic status, malnutrition, cirrhosis, HIV infection, diabetes mellitus, underlying malignancy, and ambulatory peritoneal dialysis are risk factors for tuberculous CA [28–30]. Lymphatic filariasis causes severe inflammatory reaction in lymphatic vessels leading to lymphedema and CA [31]. Infection with *Mycobacterium avium*-*intracellulare* has been reported to cause CA in AIDS patients [32]. A recent systematic review showed that infections with mycobacterial species, as MAI infection and tuberculosis, contribute to 10% of all cases of atraumatic CA [17].

#### 5.1.5. Inflammatory

A variety of inflammatory causes have been reported to be associated with CA. Both acute pancreatitis and chronic pancreatitis have been associated with CA [33]. Two mechanisms have been proposed to play a role in the development of CA by pancreatitis, which are compression of lymphatic channels or direct damage by pancreatic enzymes [33]. Fibrosing mesenteritis is rare benign process that involves inflammation, fat necrosis, and fibrosis of the mesentery [34] has been also reported to cause CA [17]. Other rare inflammatory causes include idiopathic retroperitoneal fibrosis, [35] sarcoidosis, [36] systemic lupus erythematous, [37] peritoneal dialysis, [38] and hyperthyroidism [39].

#### 5.1.6. Other Causes

Constrictive pericarditis has been also reported to cause CA [40, 41]. It causes impaired lymph drainage with thoracic duct dilatation and hypertension leading to an increase in hepatic venous pressure, thereby increasing lymph production [10]. Congestive heart failure can also cause CA by increasing formation of and impaired lymphatic drainage [12, 42, 43]. CA may develop as a result of heart failure secondary to thyrotoxic cardiomyopathy and resolve promptly if treated appropriately [44]. The nephrotic syndrome has been reported for unknown mechanism to cause chylous effusions including CA [45, 46]. A study including 90 patients with nephritic syndrome and ascites, showed that 45% of those who underwent paracentesis (16/35) had CA. However, the diagnosis was based on detection of opalescent effusion rather than by checking the triglyceride level of peritoneal fluid [47].

Celiac disease and Whipple's disease can cause CA due to mesenteric node hyperplasia [48]. Calcium channel blockers have also been implicated as a cause of CA in patients undergoing peritoneal dialysis [49–51]. In addition, sirolimus, in renal transplant setting, has also been reported to cause CA [52].

### 5.2. Traumatic CA (Table 1)

#### 5.2.1. Postoperative

Surgical interventions are well-known causes of CA secondary to direct lymphatic vessels injury. CA can occur as early as 1 week after abdominal surgery because of disruption of the lymphatic vessels or as late as several weeks to months because of adhesions or extrinsic compression of lymphatic vessels [1]. A retrospective study over a 2-year period of a cohort including 1,103 oncological patients undergoing abdominal surgical procedures revealed 1.1% incidence of postoperative chylous ascites. 7.4% of patients who underwent retroperitoneal, esophageal, gastric, or cytoreductive surgeries developed CA [53]. Other surgical procedures that can result in CA include aortic and abdominal aneurysm repair [54], retroperitoneal lymph node dissection [55], inferior vena cava resection [56], catheter implantation for peritoneal dialysis [38], distal splenorenal shunts [57], small bowel transplantation [58], liver transplantation [59], choledochal cyst excision [60], pancreaticoduodenectomy [61], anterior spinal surgery [62], laparoscopic surgeries including Nissen fundoplication [63], Roux-en-Y gastric bypass [64], adrenalectomy [65], cholecystectomy [66], and donor nephrectomy [67].

#### 5.2.2. Radiotherapy

Abdomen and pelvic radiation is a common cause of CA [2]. In a review done at Mayo clinic involving 207 patients who received whole abdomen irradiation for gynecologic malignancies, a 3% incidence of CA was reported [68]. Irradiation to abdomen can cause fibrosis of lymphatic vessels within small bowel and mesentery leading to obstruction and extravasation of lymph [69] which is typically observed after a mean of 12 months after radiation therapy [70].

#### 5.2.3. Noniatrogenic Causes

In contrast to direct injury of lymphatic vessels during surgery, blunt abdominal trauma causes CA through hyperextension and hyperflexion leading to rupture of lymphatic vessels and lymph leakage [71]. Penetrating abdominal trauma has also been reported to cause CA [72]. The battered child syndrome, which can lead to blunt abdominal trauma, accounts for approximately 10% of cases of CA in children [73]. Therefore, it is very important to exclude this diagnosis in any child presenting with CA.

## 6. Complications of Chylous Ascites

Loss of chyle into peritoneal cavity can lead to serious consequences because of the loss of essential proteins, lipids, immunoglobulins, vitamins, electrolytes, and water. While repeated therapeutic paracentesis provides relief from symptoms, the nutritional deficiency will continue to persist or deteriorate unless definitive therapeutic measures are instituted to stop leakage of chyle into the peritoneal space. In fact, in postoperative settings, this may cause increased mortality [74]. Therefore, it is very important to provide adequate nutritional support replenishing fluid loss, vitamin deficiencies, and electrolyte loss while specific therapeutic measures are planned.

In addition, continued loss of lymphocyte-rich lymph into the peritoneal space and enormous loss of protein in gastrointestinal tract lead to hypogammaglobulinemia and therefore increased susceptibility to infection [75]. Prolonged thoracic duct drainage has been used previously to induce immunosuppression in several diseases including rheumatoid arthritis and myasthenia gravis [75].

The bioavailability of certain drugs could be drastically impaired in the presence of significant chyle leak. There are reports of this phenomenon in patients with chylothorax-causing subtherapeutic digoxin [76], amiodarone [77], and cyclosporine [78] levels in the serum. Sequestration of drugs in chyle should be recognized early, to prevent subtherapeutic plasma levels in patients undergoing drainage of CA.

## 7. Evaluation and Diagnosis

The diagnostic approach of CA consists of first suspecting the diagnosis, then confirming the presence of chyle in the peritoneal cavity, and finally determining the underlying abnormality. A careful history, physical examination, and diagnostic paracentesis are the key in the initial evaluation of any patient presenting with ascites.

### 7.1. Clinical Findings

Progressive and painless abdominal distention (81%) and nonspecific pain (14%) are the most common presenting symptoms in CA, occurring over a course of weeks to months depending on the underlying cause [17]. Patients who have undergone abdominal or thoracic surgery may present with an acute onset of CA [2]. Patients may also present weight gain and dyspnea resulting from increased abdominal girth [1]. Other features include weight loss, anorexia, malaise, steatorrhea, malnutrition, enlarged lymph nodes, fevers, and night sweats [2, 3, 11]. However, most often the diagnosis of CA is not suspected before performing a diagnostic paracentesis [1].

Physical signs that may be present on examination include ascites, pleural effusions, lower extremity edema, lymphadenopathy, cachexia, temporal wasting, and hernias [1]. Other findings depend on the underlying cause.

### 7.2. Laboratory Findings

Abdominal paracentesis is the most important diagnostic tool in evaluating and managing patients with ascites. In contrast to the yellow and transparent appearance of ascites due to cirrhosis and portal hypertension, chyle typically has a cloudy and turbid appearance (Table 2). This should be distinguished from pseudochylous ascites, in which the turbid appearance is due to cellular degeneration from infection or malignancy without actually containing high levels of triglycerides [79]. Depending on the clinical suspicion, ascitic fluid should be sent for cell count, culture, Gram stain, total protein, albumin, triglyceride levels, glucose, lactate dehydrogenase, amylase, and cytology [80]. The serum to ascites albumin gradient (SAAG) should be calculated to determine if the ascites is related to portal hypertension or other causes [80]. The triglyceride levels in ascitic fluid are very important in defining CA. Triglyceride values are typically above 200 mg/dL, although some authors use a cutoff value of 110 mg/dL [1, 3]. A tuberculosis smear and culture and adenosine deaminase activity (ADA) should be performed in selected cases when tuberculosis is suspected [1]. ADA has high sensitivity and specificity in the diagnosis of tuberculous peritonitis [81]. In contrast, its utility in populations with high prevalence of cirrhosis such as the United States is limited [82]. The diagnosis of tuberculous peritonitis usually requires a peritoneal biopsy via laparoscopy [83].

Standard blood tests, including a complete blood count, electrolytes, liver tests, total protein, albumin, lactate dehydrogenase, triglycerides, cholesterol, amylase, and lipase should be performed. Additional testing should be based upon the clinical setting [84].

### 7.3. Imaging Studies

#### 7.3.1. Computed Tomography (CT)

Chyle has a water density appearance on CT which can be readily distinguished from acute hemorrhage in the setting of trauma [2]. CT of the abdomen is useful in identifying pathologic intra-abdominal lymph nodes and masses. In the setting of postoperative or traumatic causes of CA, it also helps in determining the extent and localization of fluid, particularly, if there is a suspicion of thoracic duct injury [1, 2]. Other finding on CT might suggest that CA is formation of fluid-fluid level [85]. Another CT technique reported is the direct opacification of the thoracic duct with oral fat emulsions [86].

#### 7.3.2. Lymphoscintigraphy

Lymphoscintigraphy allows functional assessment of lymphatic transport and so can be used to detect abnormal lymphatic drainage in CA. It is useful for detecting patients for surgery and assessing the effect of treatment [87–89]. It can be used when lymphangiography is contraindicated [2]. Its advantages include no adverse effects, no contraindications, and the ability to perform repetitive studies. The technical challenges of this technique and its rare implementation may make it an unfavorable diagnostic modality [2].

#### 7.3.3. Lymphangiography

Lymphangiography use has been declining with the availability of noninvasive imaging, but it remains the gold standard in defining cases of lymphatic obstruction. It has been successfully used to detect abnormal retroperitoneal nodes, leakage from dilated lymphatics, fistulization, and patency of the thoracic duct [11, 90, 91].

In addition, lymphangiography is also used in treating patients with chyle leakages who are resistant to conservative approach [92]. However, it is associated with complications including contrast hypersensitivity, tissue necrosis, fat embolism, and even transient lymphedema and CA [87, 93].

### 7.4. Laparoscopy

Ascites of unknown etiology is a common indication for laparoscopy in patients with ascites especially when tuberculosis or malignancy is suspected [2]. It was reported to be perhaps the best and most definitive method to diagnose intestinal lymphangiectasia [20].

### 7.5. Laparotomy

Early reoperation has been recommended for postoperative CA to address the underlying cause as well as for providing treatment [94]. It is advocated to do combined pre- and intraoperative lymphangiography to facilitate successful treatment of postoperative CA [95].

## 8. Management of CA

Few studies have addressed the best treatment regimens for CA [2]. Nutritional regimens and pharmacological and surgical therapies exist but there is still a lack of a clear consensus on the optimal management of CA [96]. Treatment of the underlying cause is an important initial step in managing these patients. In most cases, particularly, in patients with infectious, inflammatory, or hemodynamic cause, this will result in resolution of symptoms and of the ascites [1].

### 8.1. Medical Treatment

Medical management of CA is based on the theory that decreasing chyle flow will allow for spontaneous closure of the chyle leak [96]. However, there is no precise, functional method for monitoring the response to therapy [2].

#### 8.1.1. Dietary Therapy

Based on the limited studies and no clear consensus, it is a reasonable approach for patients in whom the cause was not found or for those who did not respond to treatment of the underlying cause to recommend the nutritional therapy. Although it is common in practice to recommend bowel rest and dietary modification, enteral feedings, or the use of total parenteral nutrition (TPN), definitive evidence supporting one nutrition therapy over another does not exist [96]. Goal of nutrition therapy is to decrease production of chyle, replace fluid and electrolytes, and maintain or improve nutrition status [97].

A reasonable approach is to recommend a high-protein and low-fat diet with MCT. Dietary restriction of LCT prevents their conversion into monoglycerides and FFA, which are transported as chylomicrons to the intestinal lymph ducts. In contrast, MCT are absorbed directly into intestinal cells and transported as FFA and glycerol directly to the liver via the portal vein. Thus, a low-fat diet with MCT supplementation reduces the production and flow of chyle [98]. Patients with advanced cirrhosis, MCT oil should not be used as narcosis and coma may occur. Such patients should be managed with a low-sodium diet and diuretics such as spironolactone [99].

Patients who do not respond to the above measures should have bowel rest to reduce lymph flow and be started on TPN [2]. TPN is theoretically superior to any enteral feedings because the bowel is bypassed. The presence of intraluminal water alone has been shown to increase thoracic duct lymph flow [2]. TPN along with somatostatin or octreotide can relieve the symptoms and rapidly close the fistula in patients with CA [100]. This approach appears to be an effective therapy for the treatment of CA caused by various disorders [101].

#### 8.1.2. Pharmacology

There are other medical measures which have been described in literature as either case reports or small observational studies. Orlistat, a reversible inhibitor of gastric and pancreatic lipases, was reported to minimize ascites and triglyceride levels in ascitic fluid in a patient with CA due to cirrhosis [102]. Case reports have suggested that both somatostatin and octreotide either alone or in combination with TPN are effective in the management of CA due to different causes [67, 101, 103–106]. The mechanism may involve inhibition of lymph fluid excretion through specific receptors found in the normal intestinal wall of lymphatic vessels [107]. In case reports, a promising treatment, etilefrine, a sympathomimetic drug, was shown to cause resolution of postesophagectomy chylous effusions [108].

### 8.2. Abdominal Paracentesis

In patients with symptomatic ascites, a therapeutic paracentesis should be performed to relieve symptoms and could be repeated as needed [1]. Unless the patient has cirrhosis, the replacement of albumin to prevent postparacentesis circulatory dysfunction is not recommended. Repeated large-volume paracentesis is a reasonable option for patients who have end-stage disease not amenable to medical or surgical treatment.

### 8.3. Transjugular Intrahepatic Portosystemic Shunt (TIPS)

The use of TIPS to successfully treat CA has been reported in patients with cirrhosis and CA resistant to conservative therapy and who have reasonable liver function [109–111]. However, the placement of TIPS in a patient with cirrhosis is associated with significant problems so patients must be selected carefully.

### 8.4. Peritoneovenous Shunting

In the past, peritoneovenous shunts (LeVeen or Denver shunts) were considered options for patients who were refractory to medical therapy and poor candidates for surgery. However, these shunts were associated with a high rate of serious complications, such as sepsis, disseminated intravascular coagulation, hypokalemia, small bowel obstruction, and risk for air embolism, and are thus seldom used [112]. In addition, the high viscosity of the chyle results in a high rate of shunt occlusion in majority of the cases [3, 94, 113].

### 8.5. Angiography

In addition, to make diagnosis, lymphangiography with or without embolization is another promising technique which has been described for the literature in the treatment of postoperative CA when conservative therapy fails [90, 92, 114].

### 8.6. Surgical Treatment

If the above conservative management is not successful in treating CA, surgical intervention may be beneficial especially in patients with postoperative, neoplastic, and congenital causes [2]. Preoperative lymphangiography or lymphoscintigraphy is helpful in identifying the anatomical location of the leakage or the presence of a fistula [3]. Laparotomy is also essential in the diagnosis and management of acute chylous peritonitis. In a review where all patients were initially treated conservatively with dietary therapy, surgery (fistula closure, bowel resection, or insertion of a peritoneovenous shunt) was performed in patients who failed conservative therapy (66%). Closure of a retroperitoneal fistula, when present, was the most successful operation [11]. However, in postoperative CA, surgical reinterventions are associated with significant incidence of morbidity and mortality [115]. In addition, surgery may occasionally fail to identify the leak. Some promising new techniques (e.g., use of octreotide, etilefrine, or angiography), which—alone or in combination with well-established conservative measures—may have the potential to avoid surgical reinterventions [115].

In addition, to prevent postoperative CA, it was found that the milk test is a safe and effective method following pancreatectomy [116]. In children, fibrin glue application for control of lymph leakage is also effective in prevention and management of postoperative CA [117] as well as management of congenital CA [118].

## 9. Conclusion

In summary, CA is a relatively uncommon disorder. Malignancy and cirrhosis are the leading causes of this condition in adults. In contrast, congenital abnormalities of the lymphatic system and trauma are common causes in children. Paracentesis with confirmation of elevated triglyceride is considered to be the gold standard for diagnosis of CA. In a cirrhotic patient, unless there is a strong suspicion of malignancy, there is no need for unnecessary and invasive diagnostic tests to rule out a malignant cause. Treatment of the underlying cause is an important initial step in managing these patients. Conservative approach includes the use of a low-fat diet, MCT intake, paracentesis, TPN, and somatostatins. Other treatment options for resistant cases include TIPS, surgical exploration, and peritoneovenous shunt. However, some promising new techniques such as use of etilefrine or percutaneous embolisation of cisterna chyli are waiting further evaluation.

## Figures and Tables

**Table 1:** Etiological classification of chylous ascites.

Atraumatic [2]		Traumatic
**(I) Neoplastic **	**Cardiac**	**(I) Iatrogenic **
Solid organ cancers	Constrictive pericarditis	**(A) Surgical**
Lymphoma	Congestive heart failure	Abdominal aneurysm repair
Sarcoma	**Gastrointestinal **	Retroperitoneal lymphadenectomy
Carcinoid tumors	Celiac sprue	Placement of peritoneal dialysis catheter
Lymphangioleiomyomatosis	Whipple's disease	Inferior vena cava resection
Chronic lymphatic leukemia	Intestinal malrotation	Pancreaticoduodenectomy
**(II) Diseases**	Small bowel volvulus	Vagotomy
**(A) Congenital **	Ménétrier disease	Radical and laparoscopic nephrectomy
Primary lymphatic hypoplasia	**Inflammatory**	Nissen fundoplication
Klippel-Trenaunay syndrome	Pancreatitis	Distal splenorenal shunts
Yellow nail syndrome	Fibrosing mesenteritis	Laparoscopic adrenalectomy
Primary lymphatic hyperplasia	Retroperitoneal fibrosis	Gynecological surgery
Lymphangioma	Sarcoidosis	**(B) Nonsurgical**
Familial visceral myopathy	Systemic lupus erythematosus	Radiotherapy
**(B) Acquired**	Behçet's disease	**(II) Noniatrogenic**
**Cirrhosis**	Peritoneal dialysis	Blunt abdominal trauma
**Infectious**	Hyperthyroidism	Battered child syndrome
Tuberculosis	Nephrotic syndrome	Penetrating abdominal trauma
Filariasis	**Drugs**	Shear forces to the root of the mesentery
*Mycobacterium avium* in AIDS	Calcium channel blockers	**(III) Idiopathic **
Ascariasis	Sirolimus	Rule out lymphoma

**Table 2:** Characteristics of ascitic fluids in chylous ascites (adapted from C$\overset{´}{\text{a}}$rdenas and Chopra) [1].

Color	Milky and cloudy
Triglyceride level	Above 200 mg/dL
Cell count	Above 500 (lymphocytic predominance)
Total protein	Between 2.5 and 7.0 g/dL
SAAG	Below 1.1 g/dL∗
Cholesterol	Low (ascites/serum ratio < 1)
Lactate dehydrogenase	Between 110 and 200 IU/L
Culture	Positive in selected cases of tuberculosis
Cytology	Positive in malignancy
Amylase	Elevated in cases of pancreatitis
Glucose	Below 100 mg/dL

IU: international units; SAAG: serum-ascites albumin gradient.

∗Is elevated above 1.1 g/dL in CA secondary to cirrhosis.
